# Colorado State Veterinary Medical Association

**Published:** 1897-09

**Authors:** D. P. Frame

**Affiliations:** Secretary; Colorado Springs, Col.


Secretary.

### COLORADO STATE VETERINARY MEDICAL ASSOCIATION.

The regular semi-annual meeting was called to order at Denver by Dr. Sol. Bock, President, in the chair, July 22, 1897. The following members answered to roll-call: Drs. Solomon Bock, F. M. Hunt, Emil Pouppirt, of Denver, and Dr. D. P. Frame, of Colorado Springs. Dr. Charles Lamb, of Denver, came in later. Dr. M. R. Trumbower, of the U. S. V. M. A., was present as a welcome visitor. Minutes of the last meeting were read and approved.

Dr. Frame made a verbal report of the progress and result of the proposed veterinary bill in the last Legislature. The bill was reported favorably by the committees of both branches of the Assembly, and was on the calendar for passage, but was not reached in time for action.

The applications for membership in the Association of M. J. Dunleavy, M.D.C., of Chicago Veterinary College, and Dr. E. Bovett, were presented by Dr. Bock, and seconded by Dr. Hunt. They were elected to membership.

A letter from Dr. A. T. Peters, of Lincoln, Neb., was read, requesting the influence of this Association in securing the next annual meeting of the U. S. V. M. A. in the West. The Secretary was directed to reply to this, assuring Dr. Peters of our support.

Dr. E. Bovett came in and was introduced to the members present.

Dr. Pouppirt nominated Dr. Lamb for Treasurer of the Association; seconded by Dr. Hunt. Dr. Lamb was elected Treasurer.

Dr. Charles Gresswell, the regular essayist for this meeting, being absent, the members indulged in a general discussion of rare and interesting cases, with treatment of the same, and in this way spent a very enjoyable and profitable afternoon.

The next meeting will be called January 4, 1898. Adjourned to meet at that time.

D. P. FRAME, M.D.C.,
Colorado Springs, Col. Secretary.

